# Clinical and paraclinical features distinguish fatigue from depression-predominant phenotypes in multiple sclerosis

**DOI:** 10.1038/s41598-025-03079-4

**Published:** 2025-05-21

**Authors:** Yavor Yalachkov, Kimberly Koerbel, Johannes Gehrig, Marion Hug, Iris-Katharina Penner, Michelle Maiworm

**Affiliations:** 1 Goethe University, University Medicine, Department of Neurology, Schleusenweg 2-16, 60528 Frankfurt am Main, Germany; 2 Department of Neurology, Bern University Hospital, Inselspital, University of Bern, Bern, Switzerland

**Keywords:** Multiple sclerosis, Fatigue, Depression, Cognitive deficits, EDSS, SDMT, Immune phenotypes, Multiple sclerosis, Neuroimmunology

## Abstract

In clinical routine, it might be difficult to distinguish between fatigue and depression in Multiple sclerosis (MS). We investigated in two independent observational cohort studies which clinical and paraclinical features distinguish patients reporting fatigue but no depressive symptoms from those suffering from depressive symptoms but having no relevant fatigue. In Study 1, *n* = 156 MS patients underwent fatigue, depression, cognitive screening and flow cytometry measurements. In Study 2, *n* = 54 MS patients performed a comprehensive neuropsychological evaluation. Patients reporting predominantly fatigue symptoms were older, had a lower information processing speed and higher EDSS compared to those scoring high on depression. CD3 + CD4 + T-helper cells count distinguished patients with depressive symptoms from those with neither depression nor fatigue. Study 2 demonstrated similar results. Additionally, progressive MS was associated with fatigue. From the whole neuropsychological test battery only SDMT distinguished fatigue from depressive phenotypes with the latter performing better. In both studies the groups did not differ regarding any other baseline variable including disease activity. Older age, slow information processing, worse physical disability and progressive disease course are distinguishing features of patients reporting more fatigue than depressive symptoms. Higher peripheral inflammatory cell counts seem to characterize depression.

## Introduction

Fatigue and depression are common symptoms in multiple sclerosis (MS)^1,2^, considerably significant for the well-being of the patients and with a crucial economic burden^3–6^. While many patients remain untreated for those symptoms^7^, depression and fatigue might persist even when effective disease-modifying treatment (DMT) is applied^8,9^. Differentiating between them in the everyday clinical routine can be difficult. Often patients report similar, overlapping symptoms such as lack of energy, tiredness or low task performance, which might be explained by both fatigue and depression. Moreover, they seem to share at least some common pathophysiological mechanisms^10,11^. Combined with the usually very short time for clinical consultations, there is a significant chance that if detected, depressive symptoms might be mistaken for fatigue and vice versa. While current therapeutic concepts for depression in MS encompass both pharmacological (e.g., SSRI medication^12^) and non-pharmacological (e.g. cognitive behavioural therapy^13^) options, there are only limited therapeutical tools for fatigue. Exercise and behavioural therapeutic interventions might alleviate fatigue symptoms but there is no solid evidence on pharmacological treatments^14^. Identifying clinical and pathophysiological distinctive features of depression vs. fatigue symptoms might facilitate more specific treatment options.

Ideally, the distinction between a predominantly fatigue-associated vs. primarily depression-driven phenotype would allow also for a more sensitive and specific recognition of MS-related symptoms, better clinical evaluations and the development of patient- and problem-tailored treatments. To this end, we performed a study (Study 1) based on real-life routine data and investigated which clinical and paraclinical variables might be able to distinguish between (1) MS patients reporting mainly depressive symptoms, (2) MS patients predominantly complaining about fatigue, (3) MS patients reporting both symptoms and (4) MS patients with neither depression nor fatigue. To validate our results and to investigate the full cognitive profile of those groups, we repeated the analysis in a second study (Study 2), where a comprehensive neuropsychological test battery was applied. In a final step, we evaluated whether motor and cognitive fatigue correlate differently with the identified clinical features.

## Materials and methods

Both Study 1 and Study 2 were performed in accordance with The Code of Ethics of the World Medical Association (Declaration of Helsinki) for experiments involving humans and were approved by the local ethics committee of the University Hospital Frankfurt. In Study 1 all data were acquired during clinical routine and analyzed retrospectively. The retrospective analysis of anonymized clinical data was approved and the need for informed consent was waived by the local ethics committee of the University Hospital Frankfurt at the Goethe University Frankfurt (Nr. 2024-661). In Study 2, study-specific written informed consent was obtained before enrollment. All patients were diagnosed according to the revised 2017 McDonald criteria (Thompson et al., 2018).

### Study 1

#### Study population

The population in Study 1 encompassed MS patients who underwent fatigue, depression, cognitive screening, clinical and lab testing as a part of the clinical routine at the Department of Neurology, Goethe University Frankfurt 01/2023-10/2023. Demographic and clinical characteristics were extracted from the database of the hospital. Physical disability was quantified by the Expanded Disability Status Scale (EDSS) and was always assessed by certified EDSS raters. Patients with the diagnosis of either CIS or RRMS according to the 2017 revision of the McDonald criteria were pooled into one category (“CIS/RRMS”). Patients with primary or inactive secondary progressive MS (SPMS) were categorized in “PMS” (progressive MS). DMTs were pooled into the categories “none”, “platform” (interferon-beta, glatiramer acetate), “oral” (teriflunomide, dimethyl fumarate, S1RP-modulators, cladribine), “natalizumab” and “anti-CD20 antibodies” (rituximab, ocrelizumab, ofatumumab). Disease activity in the last 6 months was defined by having either a clinical relapse or new, larger or contrast-enhancing lesions in the MRI imaging.

#### Neuropsychological testing

We employed Quick Inventory Depression Symptomatology (QIDS-SR16, a self-report questionnaire); Fatigue Scale for Motor and Cognitive Functions (FSMC, a questionnaire measuring motor and cognitive as well as total fatigue); and the Symbol Digit Modalities Test (SDMT, gold standard for screening for cognitive deficits in MS; it measures mainly information processing speed impairment; oral version). The cut-off for at least mild depression in QIDS-SR16 was 6 points. Scoring at least 43 points on FSMC classified as having at least mild fatigue. Subscale-specific cut-offs (motor and cognitive fatigue) were ≥ 22 points. To standardize the raw scores and allow for comparison of SDMT performance between patients of different age and educational levels, z-scores were calculated using normative data^15^.

#### Laboratory parameters

Laboratory and flow cytometry panel data were acquired from blood samples drawn for routine clinical monitoring. The following parameters were determined: C-reactive protein (CRP); white blood cells count (WBC) and lymphocyte count; flow cytometry: CD19 + and CD20 + B-cells, CD3 + and CD3 + HLA-DR + T-cells, CD3 + CD4 + T-helper cells, CD3 + CD8 + cytotoxic T-cells, CD3 + CD8 + CD38 + activated cytotoxic T-cells, CD3-CD16 + CD56 + natural killer cells (NK-cells) as well as CD4/8 ratio.

#### Statistical analysis

Based on the results from the QIDS-SR16 and FSMC testing, patients were grouped in reporting depressive symptoms only (QIDS-SR16 ≥ 6 and FSMC ≤ 42), fatigue symptoms only (QIDS-SR16 ≤ 5 and FSMC ≥ 43), both depressive and fatigue symptoms (QIDS-SR16 ≥ 6 and FSMC ≥ 43) or neither depressive nor fatigue symptoms (QIDS-SR16 ≤ 5 and FSMC ≤ 42). One-way analysis of variance (ANOVA) was employed to test for differences between the four groups with regard to each of the parameters in the study. Post-hoc t-tests were computed where relevant. For categorical variables, chi-square tests of independence were used to test for differences between the four groups. The full list of the computed measures is reported in Table 1. In the final step, we entered variables exhibiting significant effects in the ANOVA or in the chi-square tests into a multinomial logistic regression model which tested for their utility as predictors of predominant self-reported symptomatology (depression only vs. fatigue only vs. both vs. none).

**Table 1:** Based on the results from the QIDS-SR16 and FSMC testing in study 1, patients were grouped in reporting depressive symptoms only (QIDS-SR16 ≥ 6 and FSMC ≤ 42), fatigue symptoms only (QIDS-SR16 ≤ 5 and FSMC ≥ 43), both depressive and fatigue symptoms (QIDS-SR16 ≥ 6 and FSMC ≥ 43) or neither depressive nor fatigue symptoms (QIDS-SR16 ≤ 5 and FSMC ≤ 42).).

		*N*		Mean	Std. Deviation		*p*-value
Age (years)	Depression only	13		30.08	8.53		**< 0.001**
	Fatigue only	25		46.12	12.18		
	None	40		37.53	12.12		
	Both	70		42.87	13.22		
SDMT (z-score)	Depression only	13		0.22	0.87		**< 0.001**
	Fatigue only	24		− 0.99	1.34		
	None	40		0.15	1.08		
	Both	69		− 0.88	1.40		
EDSS	Depression only	13		1.54	1.35		**< 0.001**
	Fatigue only	25		3.48	1.87		
	None	39		1.73	1.56		
	Both	70		3.27	1.55		
QIDS-SR16	Depression only	13		8.08	2.69		**< 0.001**
	Fatigue only	25		3.76	1.17		
	None	40		2.63	1.76		
	Both	70		11.69	4.43		
FSMC total score	Depression only	13		32.38	5.06		**< 0.001**
	Fatigue only	25		59.80	16.56		
	None	40		28.35	6.38		
	Both	70		70.43	14.99		
FSMC cognitive score	Depression only	13		15.54	3.31		**< 0.001**
	Fatigue only	25		27.28	9.33		
	None	40		12.60	2.93		
	Both	69		33.07	8.84		
FSMC motor score	Depression only	13		17.62	2.60		**< 0.001**
	Fatigue only	25		32.88	9.12		
	None	40		16.25	5.09		
	Both	69		36.68	7.26		
Diagnosis (n)		CIS/RRMS		PMS			0.053
	Depression only	12		1			
	Fatigue only	13		12			
	None	29		10			
	Both	44		25			
Sex (n)		female		male			0.689
	Depression only	8		5			
	Fatigue only	15		10			
	None	21		19			
	Both	45		25			
DMT (n)		none	platform	oral	NTZ	antiCD20	0.612
	Depression only	2	0	1	1	9	
	Fatigue only	5	1	2	0	17	
	None	12	2	2	2	20	
	Both	23	5	8	4	27	
Disease activity in the last 6 months (n)		no		yes			0.765
	Depression only	7		5			
	Fatigue only	16		8			
	None	20		18			
	Both	38		28			
Disease duration (years)	Depression only	13		6.14	4.50		0.084
	Fatigue only	25		11.19	8.38		
	None	37		6.75	6.50		
	Both	68		8.98	8.08		
CRP (mg/dl)	Depression only	11		0.23	0.29		0.163
	Fatigue only	20		0.16	0.29		
	None	33		0.13	0.12		
	Both	53		0.23	0.24		
White blood cell count (x 10^9^/L)	Depression only	12		6.92	2.05		**0.042**
	Fatigue only	21		6.91	2.00		
	None	35		6.09	1.98		
	Both	56		7.52	2.59		
Lymphocyte count (x 10^9^/L)	Depression only	11		2.03	0.92		**0.043**
	Fatigue only	20		1.58	0.56		
	None	33		1.54	0.56		
	Both	49		1.93	0.86		
CD19 + cells (/µL)	Depression only	10		62.36	192.44		0.290
	Fatigue only	19		22.71	73.03		
	None	27		118.76	200.48		
	Both	40		106.08	201.07		
CD20 + cells (/µL)	Depression only	10		67.23	203.06		0.346
	Fatigue only	19		33.28	91.92		
	None	27		127.35	207.39		
	Both	40		112.48	206.40		
CD3 + cells (/µL)	Depression only	9		1691.67	212.86		0.095
	Fatigue only	15		1345.27	134.29		
	None	26		1215.65	80.79		
	Both	36		1488.94	102.99		
CD3 + CD4 + cells (/µL)	Depression only	9		1126.67	84.29		**0.023**
	Fatigue only	15		866.40	94.25		
	None	26		759.65	52.26		
	Both	36		997.61	72.03		
CD3 + CD8 + cells (/µL)	Depression only	9		541.44	134.13		0.685
	Fatigue only	15		446.53	55.72		
	None	26		419.62	38.22		
	Both	36		471.67	49.77		
CD4+/CD8 + ratio	Depression only	9		2.71	0.40		0.225
	Fatigue only	16		2.06	0.21		
	None	26		2.11	0.22		
	Both	36		2.76	0.31		
CD3-CD16 + CD56 + cells (/µL)	Depression only	9		291.11	52.72		0.262
	Fatigue only	14		230.57	22.84		
	None	26		245.31	19.28		
	Both	36		298.22	26.14		
CD3 + CD8 + CD38 + cells (/µL)	Depression only	9		480.44	125.56		0.586
	Fatigue only	14		378.21	47.42		
	None	26		357.46	31.83		
	Both	36		375.47	41.27		
CD3 + HLA-DR + cells (/µL)	Depression only	9		179.33	22.98		0.844
	Fatigue only	14		148.50	17.22		
	None	26		157.85	14.02		
	Both	36		162.92	16.20		

One-way analysis of variance (ANOVA) was employed to test for differences between the four groups with regard to each of the parameters in the study. Post-hoc t-tests were computed where relevant. For categorical variables, chi-square tests of independence were used to test for differences between the four groups.

*SDMT* Symbol Digit Modalities Test, *EDSS* Expanded Disability Status Scale, *QIDS-SR16* Quick inventory of depressive symptomatology (16-item) (Self-Report), *FSMC* Fatigue Scale for Motor and Cognitive Functions, *DMT* disease-modifying treatment, *CRP* C-reactive protein.

### Study 2

#### Study population

To validate our findings from Study 1 and examine the differences in the full neuropsychological performance of the investigated groups, we re-analysed the data from a previously published work, which was focused on cognitive deficits in MS^16^. MS patients were recruited in our outpatient clinic and performed a full neuropsychological testing including depression, fatigue and cognitive assessment as well as a behavioral task and an eye-tracking paradigm. The results from the analysis of the latter two tasks have been reported elsewhere^16,17^. EDSS measurement and group membership were defined similarly to Study 1. Patients were categorized into four categories: reporting depressive symptoms only (BDI-II ≥ 9 and FSMC ≤ 42), fatigue only (BDI-II ≤ 8 and FSMC ≥ 43), both depressive and fatigue symptoms (BDI-II ≥ 9 and FSMC ≥ 43) or neither depressive nor fatigue symptoms (BDI-II ≤ 8 and FSMC ≤ 42).

#### Neuropsychological testing

The full neuropsychological test battery consisted of the Rey Complex Figure Test (RCFT), written-version of SDMT, Verbaler Lern- und Merkfähigkeitstest (VLMT, a German adaptation of the Rey Auditory Verbal Learning Test), Paced Auditory Serial Addition Test (PASAT), Trail Making Test (TMT, versions A and B), Regensburger Wortflüssigkeits-Test (RWT, a German verbal fluency test), Wortschatztest (WST, a German vocabulary test), Beck Depression Inventory-II (BDI-II). Additionally, FSMC as well as the 9-hole peg test (9HPT) and a simple reaction time task to test for alertness were applied. The full list of the computed measures from the neuropsychological tests is reported in Table 2.

**Table 2:** Based on the results from the BDI-II and FSMC testing in study 2, patients were categorized into four categories: reporting depressive symptoms only (BDI-II ≥ 9 and FSMC ≤ 42), fatigue only (BDI-II ≤ 8 and FSMC ≥ 43), both depressive and fatigue symptoms (BDI-II ≥ 9 and FSMC ≥ 43) or neither depressive nor fatigue symptoms (BDI-II ≤ 8 and FSMC ≤ 42).). Since only one patient reported depressive without any fatigue symptoms, this subcategory was removed from the further analysis. One-way analysis of variance (ANOVA) was employed to test for differences between the four groups with regard to each of the parameters in the study. Post-hoc t-tests were computed where relevant. For categorical variables, chi-square tests of independence were used to test for differences between the four groups.

		*N*	Mean	Std. Deviation	*p*-value
Age (years)	Fatigue only	17	46.76	13.68	0.06
	None	12	35.08	9.82	
	Both	25	44.24	14.41	
SDMT (z-score)	Fatigue only	17	−1.67	1.22	**0.01**
	None	12	−0.23	1.01	
	Both	25	−1.01	1.08	
EDSS	Fatigue only	16	3.22	1.46	**< 0.001**
	None	11	1.95	1.04	
	Both	22	4.05	1.38	
BDI-II	Fatigue only	17	5.06	2.11	**< 0.001**
	None	12	4.33	2.39	
	Both	25	17.04	8.22	
FSMC total score	Fatigue only	17	66.00	10.06	**< 0.001**
	None	12	30.58	6.42	
	Both	25	71.32	15.76	
FSMC cognitive score	Fatigue only	17	30.88	5.63	**< 0.001**
	None	12	14.00	4.18	
	Both	25	34.48	9.97	
FSMC motor score	Fatigue only	17	35.12	6.01	**< 0.001**
	None	12	16.58	4.12	
	Both	25	36.84	6.98	
Diagnosis (n)		RRMS	PMS		**0.04**
	Fatigue only	12	5		
	None	12	0		
	Both	15	10		
Sex (n)		female	male		0.95
	Fatigue only	13	4		
	None	9	3		
	Both	18	7		
Disease duration (years)	Fatigue only	17	9.55	9.57	0.18
	None	12	4.75	5.86	
	Both	25	10.94	10.7	
Alertness (reaction time, ms)	Fatigue only	16	371.57	167.88	0.25
	None	12	290.13	54.61	
	Both	24	358.97	135.96	
9-hole peg test (dominant hand; s)	Fatigue only	16	25.03	5.5	0.25
	None	12	22.42	9.32	
	Both	25	29.26	15.8	
9-hole peg test (non-dominant hand; s)	Fatigue only	16	25.97	5.43	0.09
	None	12	21.67	5.5	
	Both	25	30.13	14.88	
RCFT (immediate recall; z-score)	Fatigue only	16	−0.39	1.63	0.41
	None	12	−1.00	1.29	
	Both	25	−0.98	1.46	
VLMT verbal learning (z-score)	Fatigue only	17	−0.14	1.5	0.38
	None	12	0.45	0.86	
	Both	25	0.02	0.97	
VLMT proportion remembered (z-score)	Fatigue only	17	−0.39	1.21	0.27
	None	12	0.21	0.7	
	Both	25	−0.10	0.92	
VLMT free recall (z-score)	Fatigue only	16	−0.25	1.56	0.05
	None	12	0.76	0.67	
	Both	25	−0.16	1.07	
PASAT (z-score)	Fatigue only	16	−0.85	1.46	0.47
	None	12	−0.20	1.45	
	Both	24	−0.60	1.26	
TMT (version A; z-score)	Fatigue only	17	−0.69	0.87	0.95
	None	12	−0.63	0.61	
	Both	25	−0.71	0.65	
TMT (B/A; z-score)	Fatigue only	17	0.01	0.83	0.94
	None	12	0.04	0.71	
	Both	25	−0.06	0.98	
RWT (phonemic fluency; z-score)	Fatigue only	16	−0.87	0.5	0.75
	None	11	−0.71	0.75	
	Both	25	−0.82	0.52	
RWT (semantic fluency; z-score)	Fatigue only	16	−0.76	0.84	0.56
	None	12	−0.51	0.82	
	Both	25	−0.78	0.63	
Vocabulary test (z-score)	Fatigue only	15	0.05	0.8	0.86
	None	12	0.17	0.65	
	Both	25	0.16	0.63	

*SDMT* Symbol Digit Modalities Test, *EDSS* Expanded Disability Status Scale, *BDI-II* Beck Depression Inventory-II, *FSMC* Fatigue Scale for Motor and Cognitive Functions, *VLMT* Verbaler Lern- und Merkfähigkeitstest, *PASAT* Paced Auditory Serial Addition Test, *TMT* Trail Making Test, *RWT* Regensburger Wortflüssigkeits-Test.

#### Statistical analysis

Since only one patient reported depressive without any fatigue symptoms, this subcategory was removed from the further analysis. Thus, patients were grouped into reporting fatigue only vs. both depression and fatigue vs. neither depression nor fatigue. Similarly to Study 1, one-way ANOVA was employed to test for differences between the three groups with regard to each of the clinical and neuropsychological parameters in the study. Post-hoc t-tests were computed where relevant. For categorical variables, chi-square tests of independence were used to test for differences between the four groups. In the final step, we entered variables exhibiting significant effects in the ANOVA or in the chi-square tests into a multinomial logistic regression model which tested for their utility as predictors of predominant symptomatology (fatigue only vs. both vs. none).

For both studies statistical analysis was performed using IBM SPSS Statistics version 29.0.2.0 and GraphPad Prism 10.4.0. For descriptive purposes, mean and standard deviations were calculated for each parameter. All significance levels were set to *p* < 0.05. Due to the exploratory nature of our investigation, no correction for multiple comparisons was applied.

### Motor vs. cognitive fatigue

To test whether our main findings differ with regard to the two subscales of the FSMC instrument (motor vs. cognitive fatigue), we pooled together the data from the two studies and computed partial correlations between the FSMC motor/cognitive subscales with each of the variables which emerged significant from the analyses of Study 1 and 2, while controlling for the presence of self-reported depressive symptoms. Each pair of correlations (e.g. “FSMC motor subscale and age” vs. “FSMC cognitive subscale and age”) was compared using z-statistics. Finally, FSMC motor and cognitive scores were compared between the groups CIS/RRMS and PMS using one-way ANOVA.

## Results

### Study 1

In total *n* = 156 patients were recruited for the study: *n* = 105 CIS/RRMS (*n* = 103 RRMS, *n* = 2 CIS) as well as *n* = 51 PMS (*n* = 22 SPMS and *n* = 29 PPMS). More detailed description of the clinical and paraclinical parameters of the cohort is given in Table 1.

**Fig. 1:**
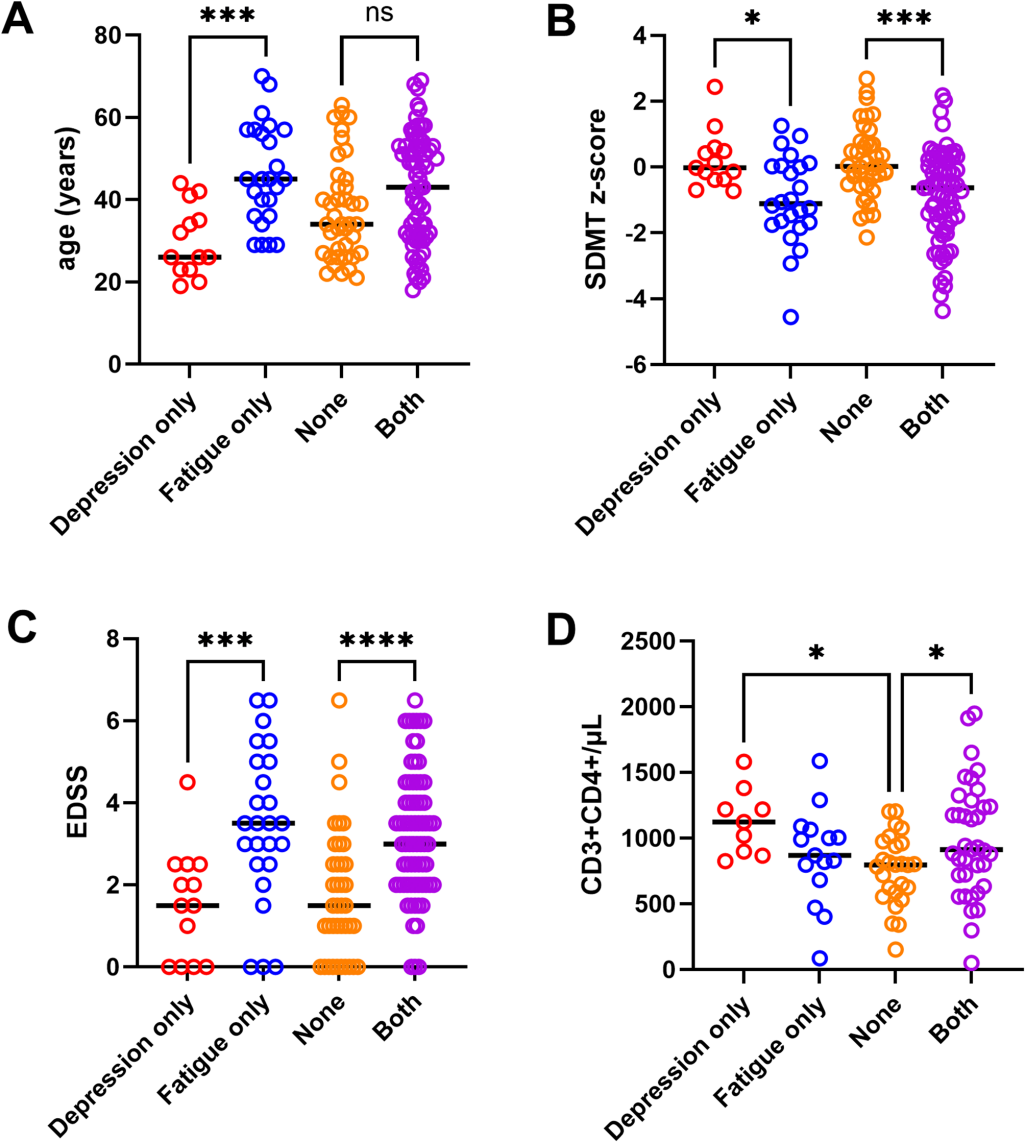
Association of clinical/paraclinical features with different MS phenotypes reporting mainly fatigue, depressive, both or none of those symptoms. Patients reporting depressive symptoms only were younger in comparison to those reporting only fatigue symptoms (**A**), had a higher information processing speed (**B**) and lower EDSS (**C**). Additionally, they had higher CD3 + CD4 + T-helper cell counts as compared to patients without depressive or fatigue symptoms, while patients with fatigue only did not differ from the latter (**D**).

Patients reporting depressive symptoms only were younger in comparison to those reporting only fatigue symptoms (30.08 ± 12.18 vs. 46.12 ± 12.18 years, post-hoc t-test *p* = 0.0005, Table 1; Fig. 1A), had a higher information processing speed (SDMT z-score 0.22 ± 0.87 vs. −0.99 ± 1.34, *p* = 0.007, Table 1; Fig. 1B) and lower EDSS (1.54 ± 1.35 vs. 3.48 ± 1.87, *p* = 0.001, Table 1; Fig. 1C). Additionally, they had higher CD3 + CD4 + T-helper cell counts as compared to patients without depressive or fatigue symptoms (1126.67/µl ± 84.29 vs. 759.65/µl ± 52.26, *p* = 0.02, Table 1; Fig. 1D), while patients with fatigue only did not differ from the latter (866.40/µl ± 94.25 vs. 759.65/µl ± 52.26, *p* = 0.36, Table 1; Fig. 1D). A significant ANOVA effect was achieved also for white blood cell count (WBC). However, this difference was driven mainly by the significant effect of patients reporting both depressive and fatigue vs. those without any symptoms (WBC: 7.52 × 10^9^/L ± 2.59 vs. 6.09 × 10^9^/L ± 1.98, *p* = 0.004, Table 1). Lymphocyte counts differed also significantly but mainly between patients with only depressive vs. those without any symptoms (2.03 × 10^9^/L ± 0.92 vs. 1.54 × 10^9^/L ± 0.56, *p* = 0.02, Table 1). Differences in the frequencies of diagnoses (CIS/RRMS vs. PMS) across the four categories of symptomatology was very close to but failed to reach significance (*p* = 0.053, Table 1).

A multinomial logistic regression was performed to create a model of the relationship between the predictor variables age, SDMT z-score, EDSS, CD3 + CD4 + T-helper cell counts, WBC and lymphocyte count and the four phenotypes. The fit between the model containing only the intercept and data improved with the addition of the predictor variables, χ^2^_(18, 83)_ = 39.16, Nagelkerke R^2^ = 0.41, *p* = 0.003. The likelihood ratio tests for each of the predictors demonstrated a significant effect for SDMT z-score only (χ^2^ = 11.39, *p* = 0.01). When comparing directly in the model patients who reported only depressive vs. no symptoms, CD3 + CD4 + T-helper cells count was the only significant predictor (*p* = 0.03). When distinguishing in the model between patients with fatigue only vs. no symptoms, SDMT z-score was the only significant predictor (*p* = 0.01). Adding diagnosis as a predictor revealed similar findings and no other predictors reached significance.

### Study 2

In total *n* = 54 patients were recruited for the study: *n* = 39 RRMS as well as *n* = 15 PMS. More detailed description of the clinical and paraclinical parameters of the cohort is given in Table 1. Patients reporting fatigue only as compared to patients without depressive or fatigue symptoms had lower SDMT z-scores (−1.67 ± 1.22 vs. −0.23 ± 1.01, *p* = 0.001, Table 2) and higher EDSS (3.22 ± 1.46 vs. 1.95, *p* = 0.02, Table 2). Diagnosis distribution differed, too (*p* = 0.04): all PMS patients reported either fatigue or depressive symptoms, while 30.8% of the RRMS patients did not report any of those (Table 2).

We repeated the multinomial logistic regression with EDSS, SDMT z-score and diagnosis as predictors. The fit between the model containing only the intercept and data improved with the addition of the predictor variables, χ^2^_(6, 48)_ = 33.54, Nagelkerke R^2^ = 0.563, *p* < 0.001. The likelihood ratio tests for each of the predictors demonstrated a significant effect for both EDSS (χ^2^ = 12.70, *p* = 0.002) and SDMT z-score (χ^2^ = 13.64, *p* = 0.001) but not for diagnosis (χ^2^ = 2.61, *p* = 0.27). When comparing patients with fatigue only vs. no symptoms directly in the model, SDMT z-score (*p* = 0.02) and diagnosis (*p* < 0.001) were significant predictors. When distinguishing patients with both symptoms from those with none, the model revealed significant contributions from EDSS only (*p* = 0.013).

Interestingly, no neuropsychological test other than SDMT distinguished among the three groups (fatigue vs. fatigue and depression vs. no symptoms). The VLMT free recall trial was close to but did not reach significance (*p* = 0.05). Adding the VLMT free recall trial performance to the multinomial logistic regression and repeating the analysis did not reveal new significant predictors.

### Pooled analysis

**Fig. 2:**
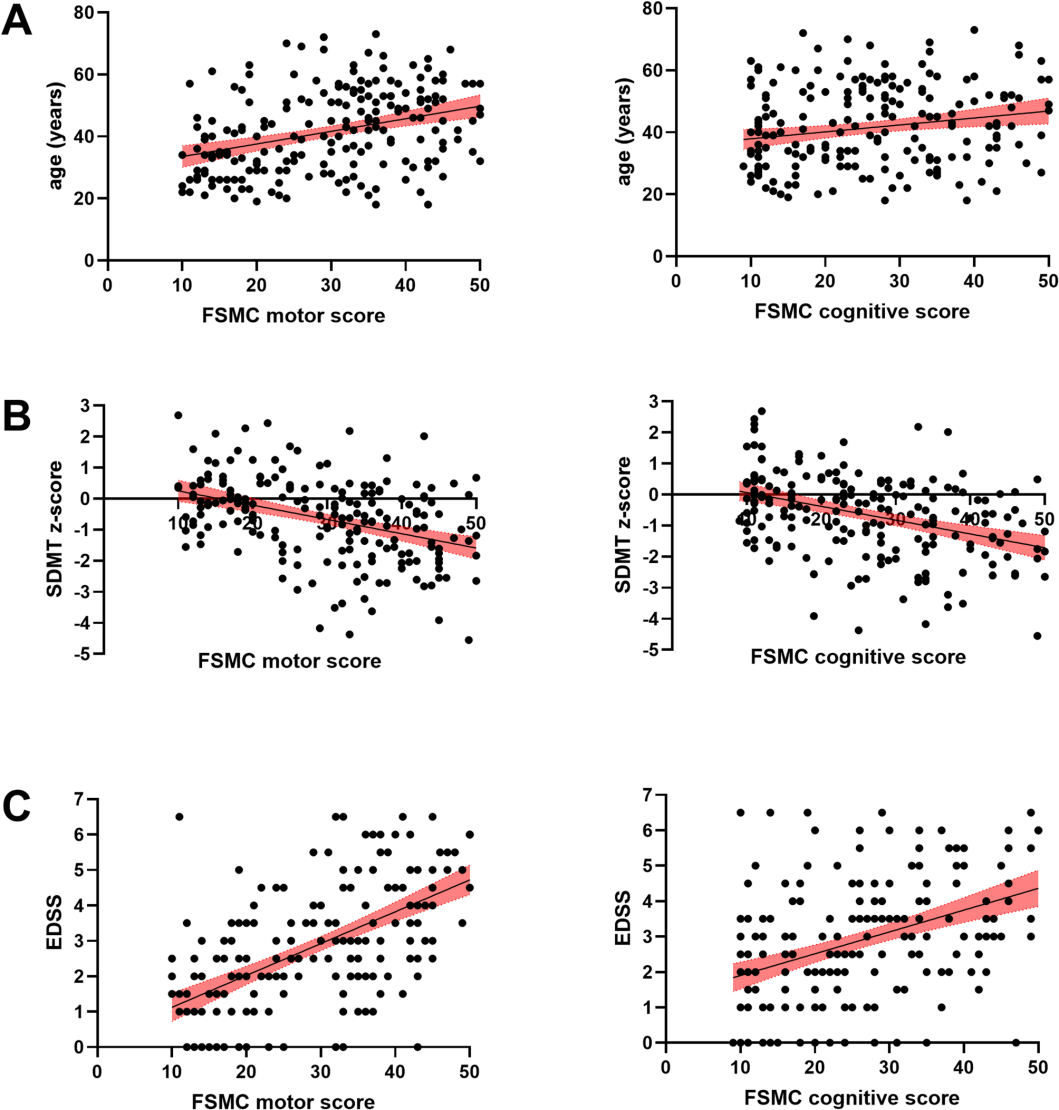
Association of clinical/paraclinical features with motor vs. cognitive fatigue scores. While age correlated with both FSMC motor and cognitive subscales, correlation between age and motor fatigue was stronger (**A**). SDMT z-score correlated with both FSMC motor and cognitive scores and those two correlations did not differ from each other (**B**). While EDSS correlated with both FSMC motor and cognitive scores, correlation between EDSS and motor fatigue was stronger (**C**).

In total the data of *n* = 207 MS patients from both studies was submitted to partial correlational analysis where reported depressive symptoms were controlled for. While age correlated with both FSMC motor (*r* = 0.375, *p* < 0.001) and cognitive (*r* = 0.211, *p* = 0.003) subscales, correlation between age and motor fatigue was stronger (z = 3.602, *p* < 0.001). SDMT z-score correlated with both FSMC motor (*r*=−0.396, *p* < 0.001) and cognitive (*r*=−0.400, *p* < 0.001) scores and those two correlations did not differ from each other (z = 0.092, *p* > 0.05). While EDSS correlated with both FSMC motor (*r* = 0.561, *p* < 0.001) and cognitive (*r* = 0.365, *p* < 0.001) scores, correlation between EDSS and motor fatigue was stronger (z = 4.633, *p* < 0.001) (Fig. 2). PMS patients scored higher on both FSMC motor (35.62 ± 10.35 vs. 26.90 ± 10.55, *p* < 0.001) and cognitive (28.59 ± 12.45 vs. 24.85 ± 11.03, *p* = 0.034) scales compared to CIS/RRMS.

## Discussion

Our findings suggest that MS patients reporting predominantly depressive vs. fatigue symptoms have distinguishing clinical and paraclinical features, which might facilitate the implementation of a more specific treatment and extend the understanding of their pathophysiology.

Patients reporting predominantly fatigue were characterized by older age, lower information processing speed and higher degree of physical disability. However, they did not differ with regard to disease activity in the last 6 months as defined by relapse or MRI dynamics and the used DMTs did not have an effect on these findings. Progression independent of relapse activity (PIRA), driven mainly by central nervous system intrinsic inflammatory and neurodegenerative processes which are different from the autoimmune inflammation arising from the periphery might be discussed as a possible factor contributing to those findings. Diagnosis (PMS vs. CIS/RRMS) was very close to reaching significance as a distinguishing feature in the first study but reached significance in the second. Here, fatigue was more strongly associated with the progressive course of the disease. Furthermore, PMS patients scored higher than CIS/RRMS on both motor and cognitive fatigue. Remarkably, in the first study duration of the disease was also close to significance. This could be an indirect hint towards the role of accumulating PIRA and smouldering inflammation, since they reveal their effects more strongly on the long term. However, this tentative explanation needs further specifically designed studies and more evidence.

“Depression only” MS phenotype was characterized by younger age, higher cognitive processing speed, lower degree of physical disability, higher T_h_ (CD3 + CD4+) cell count compared to fatigue-only MS patients and higher lymphocyte count in comparison to MS patients who reported neither depressive nor fatigue symptoms. Since peripheral inflammation is the hallmark of relapse-associated worsening (RAW) especially in the early stages of disease, the higher inflammatory cell count might be interpreted as an early subclinical disease activity which might underly depressive symptoms in MS. Any further considerations regarding this finding, however, should take into account the fact that the MS-associated inflammation is particularly complex and far from this simplified view. Furthermore, it would be of paramount interest to see whether more specific surface markers of peripheral immune cells are associated with depressive symptoms.

In a previous study we demonstrated that depressive symptoms in newly diagnosed MS patients are associated with peripheral inflammatory markers and gadolinium-enhancing lesions^18^. Furthermore, peripheral inflammation is a distinguishing characteristic of a subgroup of major depression patients who exhibit an “inflammation-driven” and particularly treatment-refractory disease course^19^. Importantly, higher lymphocyte and CD3 + CD4 + counts have been associated with current major depressive episodes^20^ corroborating our findings and suggesting that the “depression only” MS phenotype might be associated with peripheral inflammation. This might be also the explanation why this MS phenotype is apparently less affected by the disease, as suggested by the higher information processing speed, less physical disability and younger age: peripheral inflammation is more predominant in early stages of the disease and thus younger patients, who have a lesser disease burden are more probable to report predominantly depressive symptoms, while fatigue might be hypothetically an early sign of PIRA. During the further course of the disease both peripheral and CNS-intrinsic inflammation unfold their effects and both depression and fatigue accumulate.

Since SDMT performance differentiated between fatigue-only and depression-only patients, we assessed the performance in a second cohort of MS patients undergoing a full neuropsychological evaluation. Interestingly, none of the further tests differed between the groups and only the VLMT free recall trial was close to significance (*p* = 0.05). This could indicate an overlap between fatigue and cognitive deficits which is more specific for information processing speed. Alternatively, since SDMT is a rather sensitive screening tool for cognitive decline in MS^21,22^, this might suggest that fatigue-associated cognitive impairment occurs first in the information processing domain before further, more strongly pronounced deficits unfold.

It remains challenging to disentangle fatigue from depression, since “reduced energy or fatigue” is one of the ICD-11 criteria for single episode depressive disorder. However, there are MS patients who do not meet the criteria for depression but report significant amounts of fatigue. Moreover, the fatigue findings of our study seem to be not motor- or cognitive-specific since both FSMC motor and cognitive subscales correlated with age, EDSS and SDMT performance, even when controlling for reported depressive symptoms.

Our study is not without limitations. While the second cohort had the advantage of having a full neuropsychological evaluation, it was very limited in size and did not allow us to investigate depression-specific symptoms. Larger sample size as well as follow-ups would allow for a more detailed insight into the different MS phenotypes. Due to the explorative nature of this study, we did not employ a standard diagnostic interview for confirming the presence or absence of a major depressive disorder, which might could have helped stratify the results and the two cohorts. Furthermore, since the data originated from the clinical routine, only a limited flow cytometry analysis was available. A more detailed investigation of the cell subpopulations contributing to the different symptoms would allow a better understanding of the involved inflammatory processes. Last but not least, we studied self-reported symptoms of depression but did not include diagnostic interviews and clinician ratings.

Our study suggests that MS patients reporting predominantly fatigue are characterized by older age, lower information processing speed, higher degree of physical disability and possibly by a progressive disease course. MS patients reporting mainly depressive but no fatigue symptoms are younger, perform better in SDMT and are less physically impaired, while exhibiting higher peripheral inflammatory cell counts. This allows a more precise identification of fatigue- vs. depression-predominant clinical phenotypes and possibly a better understanding of the underlying pathophysiological possibilities with fatigue possibly more closely associated with CNS-intrinsic, PIRA-related processes while depression being rather linked to peripheral inflammation.

## Data Availability

Data that support the findings of this study are available upon reasonable request to the corresponding author.
